# Mathematical analysis and algorithms for efficiently and accurately implementing stochastic simulations of short-term synaptic depression and facilitation

**DOI:** 10.3389/fncom.2013.00058

**Published:** 2013-05-10

**Authors:** Mark D. McDonnell, Ashutosh Mohan, Christian Stricker

**Affiliations:** ^1^Computational and Theoretical Neuroscience Laboratory, Institute for Telecommunications Research, University of South AustraliaMawson Lakes, SA, Australia; ^2^John Curtin School of Medical Research, Australian National UniversityCanberra, ACT, Australia

**Keywords:** short term synaptic dynamics, short term depression, facilitation, stochastic simulation, stochastic synapse, vesicle site model, synaptic plasticity models, short term plasticity

## Abstract

The release of neurotransmitter vesicles after arrival of a pre-synaptic action potential (AP) at cortical synapses is known to be a stochastic process, as is the availability of vesicles for release. These processes are known to also depend on the recent history of AP arrivals, and this can be described in terms of time-varying probabilities of vesicle release. Mathematical models of such synaptic dynamics frequently are based only on the mean number of vesicles released by each pre-synaptic AP, since if it is assumed there are sufficiently many vesicle sites, then variance is small. However, it has been shown recently that variance across sites can be significant for neuron and network dynamics, and this suggests the potential importance of studying short-term plasticity using simulations that do generate trial-to-trial variability. Therefore, in this paper we study several well-known conceptual models for stochastic availability and release. We state explicitly the random variables that these models describe and propose efficient algorithms for accurately implementing stochastic simulations of these random variables in software or hardware. Our results are complemented by mathematical analysis and statement of pseudo-code algorithms.

## 1. Introduction

The release of vesicles following arrival of a pre-synaptic action potential (AP) at a synapse is inherently probabilistic (Vere-Jones, 1966; Melkonian and Kostopoulos, 1996; Branco and Staras, 2009). The amount of neurotransmitter released by each AP can also vary stochastically over time, in a manner dependent on the timing of previously arriving APs (Dobrunz and Stevens, 1997). These effects result in what is called short-term synaptic plasticity (Zucker et al., 2002; Klug et al., 2012; Regehr, 2012). It has been suggested that the short term dynamics such plasticity introduces may play an important role in information processing in the cortex (Abbott and Regehr, 2004; Branco and Staras, 2009). This has been demonstrated in studies of the influence of short-term plasticity on: gain control (Abbott et al., 1997); coding and detection mechanisms (Tsodyks and Markram, 1997; Maass and Zador, 1999); filtering effects (Matveev and Wang, 2000a; Merkel and Lindner, 2010; Rosenbaum et al., 2012); redundancy reduction (Goldman et al., 2002); information transmission (Goldman et al., 2004); membrane potential estimation (Pfister et al., 2010); attractor networks (Fung et al., 2012); and correlations in neural activity (Rosenbaum et al., 2013).

Popular mathematical models of short term synaptic plasticity effects, such as depression and facilitation, typically are expressed in term of differential equations that describe how the mean number of available and/or released vesicles changes with time in response to pre-synaptic spiking (Tsodyks and Markram, 1997; Tsodyks et al., 1998). The mean is an ensemble-average over multiple repeats of the same pre-synaptic spike train, and is often the focus of study because if the number of vesicles in the model is large, the variance across trials is small and assumed to be negligible in its impact. The consequence of this assumption is that simulations of this type of model of short term plasticity provides deterministic outcomes, in the sense that they do not produce varying outcomes if repeated trials with identical initial conditions are simulated.

However, variability in the number of vesicles available/released has also been studied mathematically (Vere-Jones, 1966), as has the covariance in the response to consecutive pre-synaptic APs (Quastel, 1997). Recently, it has been shown mathematically that explicit inclusion of the variance in models of short-term plasticity leads to significant differences in terms of frequency-dependent information transmission, in comparison with models that study only the mean (Rosenbaum et al., 2012). This mathematical finding that variance can be influential is consistent with previous simulation results (discussed in following paragraphs) that found that the mean-model underestimates post-synaptic firing rate (de la Rocha and Parga, 2005).

As well as mathematical analysis, the conceptual models of stochastic vesicle availability and release that these models are based on can also be studied by implementing stochastic simulations. We use the term “stochastic simulation” to mean a software (or, potentially, hardware) implementation that explicitly generates random or pseudo-random numbers for the purposes of simulating outcomes of a model's random variables (Gillesple et al., 1977). By doing this, repeated runs with identical initial conditions and identical external input to the model results in randomly varying outcomes, i.e., trial-to-trial variability. Such simulations have, for example, been used to study ion-channel noise and its impact on AP generation (Faisal and Laughlin, 2007).

Although the mean model described above has been used frequently, results based on stochastic simulations of short term plasticity models have also been described previously (Melkonian and Kostopoulos, 1996; Quastel, 1997; Matveev and Wang, 2000b, a; Fuhrmann et al., 2002; de la Rocha and Parga, 2005; Loebel et al., 2009; Rosenbaum et al., 2012, 2013; Scott et al., 2012; Reich and Rosenbaum, 2013) and comparisons between simulations of the deterministic and stochastic models have been shown to give rise to different outcomes in neural activity (de la Rocha and Parga, 2005; Rosenbaum et al., 2012; Scott et al., 2012).

In general, it may be important to implement stochastic simulations for synaptic connections where only a very small number of vesicles are available for release, which is often the case (Branco and Staras, 2009). In this case the mean model might be very inaccurate in scenarios where ensemble averaging across multiple repeated trials is not possible, such as in large network simulations.

As noted above, previous work has published results from stochastic simulations as a complement to mathematical analysis. However, as far as we are aware, the implementation details have not been discussed at a level of detail that will enable researchers whose primary expertise and experience is not in implementing stochastic simulations, or who have little mathematical training, to introduce trial-to-trial variation in simulations.

The primary aim of this paper is, therefore, to articulate precisely how to efficiently implement stochastic simulations that accurately reflect several of the most well-known conceptual models of vesicle availability and release processes. In our discussion, and associated pseudo-code algorithms, we assume that the algorithms would be applied under conditions where the number of vesicles available may be small, and that therefore stochastic simulation of all random variables in the conceptual models may be important. We also aim to present mathematical descriptions of key random variables that must be simulated in stochastic models, as well as relating these descriptions to existing equations describing mean numbers of vesicles. A secondary aim is to show how existing algorithms may be made more efficient and general.

As well as the usual models of release dependent depression and facilitation, the content of this paper is equally applicable to the case of release-independent depression and associated frequency dependent recovery (Fuhrmann et al., 2004; Scott et al., 2012; Mohan et al., 2013).

The paper is organized as follows. In section 2, we review conceptual models that we will use in this paper and in section 3 we mathematically introduce notation to describe the random variables implied by each conceptual model. Next, section 4 contains descriptions of correct and incorrect implementations of stochastic simulations of the conceptual models, and relates these to the random variables we described. Section 5 describes example simulation results, and shows that incorrect implementations can significantly miscalculate the number of vesicles that should be released in response to sequences of pre-synaptic AP arrivals. Finally, the conclusions drawn from our paper are summarized in section 6.

## 2. Conceptual models of short term plasticity

The first step in computational modeling is to state a conceptual model; once stated, a primary goal of computational modeling is to faithfully implement simulations of the conceptual model (Carnevale and Hines, 2005). We therefore first clearly articulate conceptual stochastic models in this section, and discuss algorithms for faithfully implementing stochastic simulations of them in the following sections. Other conceptual models exist, but the ones we consider serve to illustrate important principles that should be reflected in stochastic simulations.

### 2.1. Availability of a single vesicle following relase

In this paper we consider two conceptual “release-site” models (Sterratt et al., 2011) for short term synaptic depression, due to stochastic unavailability of a vesicle:

- **Availability Model 1:** In this model it is assumed that once a specific vesicle is released, the time at which it is next available for release is a random variable that depends only on the time since it was released. This random variable is not affected by subsequent pre-synaptic spikes.
- **Availability Model 2:** Like Availability Model 1, it is also assumed that after the vesicle is released, the time that passes before it is next available for release is a random variable. However, now if a pre-synaptic spike arrives before the vesicle becomes available, the time before the unavailable vesicle then becomes available is recalculated in a manner dependent only on the time of the latest pre-synaptic spike.

Note that these models treat a single vesicle as if it is a conserved object that switches between two states. Of course in reality the vesicle is not conserved, and a more accurate description is to say that a vesicle release site that can contain at most a single vesicle either (1) does contain a vesicle, or (2) does not contain one.

Below we show that Availability Model 1 and Availability Model 2 are mathematically equivalent, given an assumption that the random variable describing availability times is exponential. This is a standard assumption, because it provides good fits to experimental data, and therefore underpins models developed in conjunction with experimental data on short term depression (for example, Tsodyks and Markram, 1997). However, it is feasible that better fits to data might discard the exponential assumption, and in that case it would be necessary to consider how stochastic simulations need to differ for each model. As we show below for a non-exponential example (Figure 6), the two models provide significantly different outcomes.

### 2.2. Release of a single vesicle upon arrival of a pre-synaptic spike

In this paper we consider two conceptual models for the stochastic release of a single vesicle upon arrival of a pre-synaptic spike:

- **Release Model 1:** In this model it is assumed that if the single vesicle is available, then it is released with a constant probability upon arrival of a pre-synaptic spike, and this probability does not change over time.
- **Release Model 2:** In this model it is assumed that if the single vesicle is available, then it is released upon arrival of a pre-synaptic spike with a certain time-varying probability.

Release Model 1 is a classical model of probabilistic release (Vere-Jones, 1966). Release Model 2 is appropriate when a synapse is known to exhibit facilitation. Usually, based on experimental evidence (see, for example, Markram et al., 1998), the change in release probability (given availability) over time is modeled as increasing by a percentage of the current probability of non-release, whenever a pre-synaptic spike arrives (usually independently of whether a vesicle is available or released) and then decaying exponentially over time to a constant rest probability, for as long as no more pre-synaptic APs arrive (Tsodyks et al., 1998).

Release Model 2 is also appropriate when a synapse is known to exhibit a different form of short term depression to that modeled by the lack of vesicle availability. In this type of depression, known as “release-independent depression,” the probability of vesicle release (given its availability) is reduced by arriving pre-synaptic spikes independently of whether the vesicle is released, due to different mechanisms from those that cause facilitation (Fuhrmann et al., 2004). In some models, facilitation and release-independent depression are assumed to be present simultaneously (Graham and Stricker, 2008; Scott et al., 2012).

### 2.3. Combining availability and release

A single vesicle obviously cannot be released if it is not available, but it is assumed that an available vesicle remains available until released. This is the key feature of the conceptual models we study where both availability and release are modeled as stochastic.

### 2.4. Availability and release for a pool of *N* vesicles

In this paper, when we consider a conceptual model where there is a pool consisting of at most *N* vesicle release sites, each containing at most a single vesicle, we use the typical assumption that the release and availability of each single vesicle occurs independently of that in the other vesicle release sites. Note that although this model is typical, it may not always be accurate (Quastel, 1997).

### 2.5. Multiple trials of availability and release

In this paper, when we consider a conceptual model where there are *N* repeated trials for the same sequence of pre-synaptic APs, and a single vesicle in a single release site, we assume that the availability or release of the vesicle is independent for each trial.

Note that the outcome for a model where there are *N* such repeated trials is equivalent mathematically to a conceptual model where there is a pool of *N* vesicle release sites with at most a single vesicle available, for a single trial of the sequence of APs.

Since an experimental protocol is more amenable to studying repeated trials for a single release site and the same sequence of APs, we will refer to the case of *N* trials rather than *N* release sites.

### 2.6. Vesicle release sites containing multiple vesicles

The content of this paper regarding stochastic simulations can be extended to a scenario where multiple vesicles are available in a release site, and also where multiple such sites are available, potentially each with different numbers of vesicles. However, we do not discuss this further, as the most important observations are relevant to sites containing single vesicles. Further discussion of evidence for multiple release sites can be found in Loebel et al. (2009).

## 3. Random variables implied by stochastic conceptual models

The purpose of this section is to explicitly describe all random variables inherent in the conceptual models we study, since correct stochastic simulations of the models relies on correct simulation of outcomes from these random variables.

### 3.1. Availability models

There is a specific random variable that exists in both conceptual availability models: the time taken for vesicle to become available following a successful release at time *t* = *t*_s_. We label this random variable as *T*_a1_ for Availability Model 1, and as *T*_a2_ Availability Model 2.

#### 3.1.1. Availability model 1

In standard existing models, the random variable describing the time until a release site contains an available vesicle, following release of its vesicle, is exponentially distributed with a known mean, τ_a_. In this section we generalize to arbitrary positive and continuously valued distributions for the availability time. We write the probability density function describing the random variable *T*_a1_ as *f*_*T*_a1__(*T*_a1_ = *x*), and its cumulative distribution function [describing Prob(*T*_a1_ ≤ *y*)] as *F*_*T*_a1__(*y*). We introduce *P*_a,1_(*t*|*t*_s_) to describe the probability of availability at time *t*, given that the most recent successful release was at time *t*_s_. We can write

(1) $$P_{\text{a},1}(t|t_{\text{s}})=F_{T_{\text{a1}}}(t-t_{\text{s}}),t\geq t_{\text{s}}.$$

Below, we note how this probability describes a distribution of the potential times, immediately following a successful release at *t* = *t*_s_, at which the released vesicle will next become available. However, it is crucial to note that for a stochastic simulation to be faithful to Availability Model 1, the released vesicle must always be in one of two states (available or not available) and that once it switches from not-available to available, it must stay available, until released again. Ignoring this fact can lead to incorrect implementations of the conceptual model.

We now derive an expression for a conditional probability that is potentially useful in some stochastic simulation implementations. Suppose a vesicle was released at the *i*–th AP. We introduce notation for the time interval between APs, *i* and *j* as θ_*i*, *j*_ = *t*_AP, *j*_ − *t*_AP, *i*_ > 0, where *j* may be any AP after the *i*–th one. The probability that the vesicle becomes available by the *j*-th AP is Prob(*T*_a1_ ≤ θ_*i*, *j*_) = *F*_*T*_a1__(θ_*i*, *j*_), *j* = *i* + 1, *i* + 2, …. However, we also are interested in Prob(*T*_a1_ ≤ θ_*i*, *j*_|*T*_a1_ > θ_*i*, *j* − 1_) *j* = *i* + 2, *i* + 3, …, which is the probability that the vesicle does not become available before the *j* − 1–th AP, but does becomes available before the *j*–th AP. By Bayes' rule, this probability can be written as

(2) $$\begin{array}{l} \text{Prob}(T_{\text{a1}}\leq \theta_{i,j}|T_{\text{a1}}>\theta_{i,j-1})=\frac{\text{Prob}(T_{\text{a1}}\in [\theta_{i,j\;-1},\theta_{i,j}])}{\text{Prob}(T_{\text{a1}}>\theta_{i,j-1})}\\ \;=\frac{F_{T_{\text{a1}}}(\theta_{i,j})-F_{T_{\text{a1}}}(\theta_{i,j\;-1})}{1-F_{T_{\text{a1}}}(\theta_{i,j-1})} \end{array}$$

**Special Case:** For the case where *F*_*T*_a1__(*y*) = 1 − exp(−*y*/τ_a_), i.e., *T*_a1_ is exponentially distributed with mean equal to τ_a_, it is simple to derive Prob(*T*_a1_ ≤ θ_*i*, *j*_|*T*_a1_ > θ_*i*, *j* − 1_) = *F*_*T*_a1__(*t*_AP_, *j* − *t*_AP_,*j* − 1). So, in this special case, the probability of a vesicle becoming available after the *j*–th spike, given it wasn't available at the time of the *j* − 1–th spike, is independent of the time at which the vesicle actually became unavailable in the first place. This observation is actually a well known property of Poisson point processes: events in every increment of time are independent of the past history of the process. These processes have exponentially distributed inter-event distributions, as we assumed in this discussion.

#### 3.1.2. Availability model 2

A direct translation of this conceptual model implies that a random variable must be evaluated for every pre-synaptic AP that arrives while a vesicle remains unavailable. We write the time of the *k*–th pre-synaptic AP after the most recent release as *t*_AP_, *k*, where *k* = 0, 1, 2, …, *K*, *t*_AP, 0_ = *t*_s_ is the time at which the vesicle was previously released, and *K* is the number of AP arrivals before the vesicle actually becomes available. We write the random variable evaluated at the *k*–th AP as *T*_a2_, *k*.

Under Availability Model 2, we can write that if the vesicle did not become available by the *k*–th AP, then the conditional probability that a vesicle is available by time *t* is

(3) $$\begin{array}{l} P_{\text{a},\text{2}}(t|t>t_{\text{AP},k})=\text{Prob}(t_{\text{AP},k}+T_{\text{a2},k}\leq t)\\ \;=F_{T_{\text{a2}}}(t-t_{\text{AP},k}),\;t\in (t_{\text{AP},k},t_{\text{AP},k+1}], \end{array}$$

where it is assumed that each *T*_a2_, *k* is drawn independently from the same distribution with cumulative distribution function *F*_*T*_a2__(*y*).

For this model, the probability of availability by time *t*, given only the most recent release time, *t*_s_ is given by

(4) $$P_{\text{a},2}(t|t_{\text{s}})=1-(1-F_{T_{\text{a2}}}(t-t_{\text{AP},K}))\prod_{k=0}^{K-1}\times (1-F_{T_{\text{a2}}}(t_{\text{AP},k+1}-t_{\text{AP},k})),$$

which clearly in general is different from *P*_a, 1_(*t*|*t*_s_) for Availability Model 1.

This direct translation of the conceptual model to obtain *P*_a,2_(*t*|*t* > *t*_AP_, *k*) suggests a stochastic simulation implementation where a new random number is drawn for an unavailable vesicle, upon every AP arrival. However, if we can derive the cumulative distribution function of the total time to availability under this release model, *T*_a2_, a stochastic simulation that only draws a single random number upon every vesicle release is feasible. Such a random variable would have to produce *P*_a, 2_(*t*|*t*_s_) according to the above expression, and in general such a random variable is not readily obtainable. The following describes a special case where it is.

**Special case:** For an exponential distribution of *T*_a_ we can easily derive from Equation (4) that

(5) $$P_{\text{a},2}(t|t_{\text{s}})=1-\exp(-(t-t_{\text{s}})/\tau_{\text{a}}),\;t\geq t_{\text{s}}.$$

Consequently, by inspection of Equation (1), Availability Model 1 is equivalent to Availability Model 2, for exponential availability times. This equivalence can also be seen by considering Equation (2).

There are, of course, other possible models for the distribution of the release time, such as a Rayleigh or lognormal model, and it is feasible that such models may be a better fit to data than the assumed exponential model. For example, more complex models exist that describe the biophysics of vesicle generation, and how release probability depends on calcium concentration (Meddis, 1986; Sumner et al., 2002; McDonnell et al., 2008). Discussing the accuracy of simplifying such models to the phenomenological model used here is beyond the scope of this paper. In general, however, any non-exponentially distributed *T*_a_ will not lead to equivalence between Availability Model 1 and Availability Model 2.

### 3.2. Release models

There is a specific random variable that exists in both conceptual release models: the event that a vesicle is released, or not released, upon arrival of the *i*–th pre-synaptic AP at time *t* = *t*_AP, *i*_. We label this random variable as *R*(*t*_AP, *i*)_. This random variable is binary, it exists only at each AP time, it depends on the last time at which a vesicle was released, *t*_s_, and we denote its outcomes as α if a vesicle is released and as β if it is not. We denote the probability that the event α occurs at time *t*, given the vesicle is available, as *P*_r|a_(*t*). The random variable has a probability mass function, and this is given for Availability Model 1 by

(6) $$\begin{array}{l} \text{Prob}(R(t_{\text{AP},i})=\alpha |t_{\text{s}})=P_{\text{r}|\text{a}}(t_{\text{AP},i})F_{T_{\text{a}}}(t_{\text{AP},i}-t_{\text{s}});\\ \text{Prob}(R(t_{\text{AP},i})=\beta |t_{\text{s}})=1-P_{\text{r}|\text{a}}(t_{\text{AP},i})F_{T_{\text{a}}}(t_{\text{AP},i}-t_{\text{s}}), \end{array}$$

where *t*_AP, *i*_ > *t*_s_, and for Availability Model 2 by

(7) $$\begin{array}{l} \text{Prob}(R(t_{\text{AP},i})=\alpha |t_{\text{s}})=P_{\text{r}|\text{a}}(t_{\text{AP},i})P_{\text{a},2}(t_{\text{AP},i}|t_{\text{s}});\\ \text{Prob}(R(t_{\text{AP},i})=\beta |t_{\text{s}})=1-P_{\text{r}|\text{a}}(t_{\text{AP},i})P_{\text{a},2}(t_{\text{AP},i}|t_{\text{s}}). \end{array}$$

Note that in Release Model 1, *P*_r|a_ has no time dependence [i.e., *P*_r|a_(*t*_AP, *i*_) = *P*_r|a_], but this is the only difference in comparison with Release Model 2 (see Equation 1). Consequently, provided the release probability has been calculated correctly at each point in time during a simulation, there are no other differences in a stochastic simulation implementation in comparison with Release Model 2.

### 3.3. Combining availability and release

A relevant binary-valued stochastic process can be stated based on the random variables described above, namely, the process describing whether a vesicle is available at any point in time. A succinct description of this process is given in Loebel et al., (2009, Equation 4), where it is expressed in terms of a differential equation. Following the notation used in that description, we label the stochastic process as σ(*t*), and let σ(*t*) = 1 when the vesicle is available and let σ(*t*) = 0 otherwise. The process is fully described by the following equation:

(8) $$\begin{array}{l} \frac{d\sigma (t)}{dt}=-\sigma (t^{-})R(t)\delta (t-t_{\text{AP},i})\\ \;+(1-\sigma (t^{-}))\delta (t-t_{\text{AP},i}-T_{\text{a}}), \end{array}$$

where the notation ^−^ in *t*^−^ is used as shorthand to represent *t*^−^ = *t* − ∈, where ∈ is a very short time period; thus when *t* = *t*_AP, *i*_ then *t*^−^ is the time instant immediately prior to AP *i* arriving. We have assigned α = 1 and β = 0 as the possible values of the random variable *R*(*t*). During intervals of time for which σ(*t*) = 0, the right hand side of Equation (8) is just δ(*t* − *t*_AP, *i*_ − *T*_a_), and mathematically, the remaining terms in Equation (8) describe the fact that σ(*t*) can jump from 0 to 1 only at the time *t* = *t*_AP, *i*_ + *T*_a_. Similarly, Equation (8) is such that σ(*t*) can jump from 1 to 0 only when both *R*(*t*) = α = 1 and *t* = *t*_AP, *i*_, or equivalently, *R*(*t*_AP, *i*_) = 1, which means a vesicle is released when AP *i* arrives.

Note that in Loebel et al. (2009), the event where σ(*t*) jumps from 0 to 1 is stated to be modeled as a Poisson process. A Poisson process has exponentially distributed times between events, and therefore the conceptual model in Loebel et al. (2009) is in this sense the same as our Availability Model 1 with exponentially distributed *T*_a1_, with mean τ_a_. However, for an actual Poisson process, events will continue to occur for all time, not just when vesicles are currently unavailable, which is at odds with our stated conceptual model. Nevertheless, it can be inferred that in Loebel et al. (2009) that Poisson events are ignored when σ(*t*) = 1.

Does this mean that the distribution of times until a vesicle becomes available is different in each conceptual model, since in the Poisson process, the exponential time to arrival begins at the time of the previous Poisson event, whereas in Availability Model 1 begins at the most recent release time? The answer is no, due to the independence of events in Poisson processes (the same reason that Availability Models 1 and 2 are equivalent for exponentially distributed availability times). Therefore, there will be no difference when a stochastic simulation implementation of the Loebel et al. (2009) conceptual model is carried out, compared with an implementation of our Availability Model 1 with exponentially distributed arrival times. However, if the arrival times are not exponential, and the corresponding non-Poisson process replaces the Poisson process in the Loebel et al. (2009) conceptual model, the results will not be the same.

### 3.4. Deterministic mean models for availability models 1 and 2

Differential equation notation is often used to express how the mean fraction of available vesicles, *N*_a_(*t*), changes over time in two ways: either upon a spike arrival, or between spike arrivals (Tsodyks and Markram, 1997; Fuhrmann et al., 2002; Scott et al., 2012). The typical form of such expressions is

$$\frac{dN_{\text{a}}(t)}{dt}=\frac{1-N_{\text{a}}(t)}{\tau_{\text{a}}}-N_{\text{r|a}}N_{\text{a}}(t^{-})\sum_{i=1}^{K}\delta (t-t_{\text{AP},i}),$$

where *t*_AP, *i*_ is the arrival time of the *i*–th AP, out of a total of *K*, and *N*_r|a_ is the mean fraction of available vesicles released by the *i*–th AP. This differential equation can be easily solved in closed form (e.g., Tsodyks and Markram, 1997) to get

(9) $$\begin{array}{l} \;N_{\text{a}}(t)=1,\;t<t_{\text{AP},1},\\ N_{\text{a}}(t_{\text{AP},i})=(1-N_{\text{r}|\text{a}})N_{\text{a}}(t_{\text{AP},i}^{-}),\;t=t_{\text{AP},i},\\ \;N_{\text{a}}(t)=1-(1-N_{\text{a}}(t_{\text{AP},i}))\;t\in [t_{\text{AP},i},t_{\text{AP},i+1}),\\ \;\exp\left(-(t-t_{\text{AP},i})/\tau_{\text{a}}\right) \end{array}$$

where *i* = 1, …, *K*.

Note that the change over time in the fraction of trials in which the vesicle is available clearly has a dependence on both (1) the time since the most recent pre-synaptic AP and (2) on the fraction of vesicles available at the time of the most-recent pre-synaptic AP. Consequently, the deterministic mean model should be interpreted as explicitly solving for the *conditional* mean number of vesicles released at each AP arrival, *given* the number that are available for release.

**Remark 1:** It is clear that the mean model accurately reflects Availability Model 2 generally, and in the specific case stated above, assumes exponential availability times following each AP arrival. Moreover, we have discussed that Availability Models 1 and 2 are equivalent for exponentially distributed availability times, and hence the stated mean model also accurately reflects Availability Model 1 for this specific case.

**Remark 2:** The deterministic mean model does not, however, accurately reflect Availability Model 1 for non-exponentially distributed availability times, since under Availability Model 1, the fraction of trials in which a vesicle should be released, given that it is available, should be based on the trial-dependent time since a vesicle was released, not solely on the time since the most recent AP. Therefore, the right hand side of a differential equation describing the mean number of trials in which a vesicle is available should have an additional term for each AP that occurs prior to the current AP. Moreover, if each additional term describes the mean number of trials in which vesicles have not become available since the *i*–th AP, the results will potentially become increasingly inaccurate with the time elapsed since the *i*–th AP.

One possibly useful element in any extension of mathematical analysis to this case of multiple trials might be an iterative expression articulated in a different context by McDonnell et al. (2002, 2008), that can be adapted to describe the conditional probability that *u* vesicles are available across *Z* trials, even if the time they were released differs. This approach does not suggest a straightforward method for implementing a stochastic simulation, but as described by McDonnell et al. (2002, 2008), there are simple expressions for the conditional mean and variance, and these could potentially be used within a deterministic equation that describes how the mean number of trials in which a vesicle is available changes with time.

In section 5, we compare the results of stochastic simulations with results for the mean obtained from Equation (9). We also use a result for a scenario where pre-synaptic APs arrive at the synapse periodically with frequency *f* Hz so that the AP times are *t*_AP, *i*_ = *i*/*f*, *i* = 1, 2, …. In this case, it is well known that the mean fraction of vesicles available quickly decays to a constant steady state value, *N*^−^_ss_ := *N*_a_(*t*^−^_AP, *i* + 1_) = *N*_a_(*t*^−^_AP, *i*_). As shown in (Abbott et al., 1997; Matveev and Wang, 2000b), this can be obtained from Equation (9) (which hold for Availability Model 2 generally, and for Availability Model 1 with exponentially distributed availability times) to get the mean fraction of vesicles available for release just prior to a pre-synaptic spike as

(10) $$N_{\text{ss}}^{-}=\frac{\left(1-\exp\left(-1/(f\tau_{\text{a}})\right)\right)}{1-\exp\left(-1/(f\tau_{\text{a}})\right)(1-N_{\text{r}|\text{a}})}.$$

## 4. Correct and incorrect stochastic simulations, in response to pre-synaptic spike trains

We consider how a synaptic vesicle release site, containing at most a single vesicle, responds over time (*t* ≥ 0), to a sequence of *K* arriving pre-synaptic APs. A stochastic simulation implementation that is faithful to the conceptual models is one that accurately produces vesicle releases that reflect the probabilities stated in Equation (6) or in (7).

In order to carry this out, it is necessary at every time step of the simulation to have a determined state of the availability of the vesicle. In other words, the vesicle is either available or not available. It switches from available to not available in the event that it is released, and it switches from not-available to available once, and only once, in the time following its last release. Therefore, once the vesicle becomes available according to the stochastic simulation, after a time *T* since the previous release, the probability of availability that must be used within the simulation is given by

(11) $$\begin{array}{l} P_{\text{a},\text{f}}(t|t_{\text{s}})=1,\;t\geq T\\ \;0,\;t<T. \end{array}$$

This holds for both Availability Model 1 and Availability Model 2.

There are a number of parameter values that are required to be set in order to simulate a stochastic synapse model, as introduced above. These are summarized in Table 1.

**Table 1:** **List of parameter notation for single-vesicle release models**.

**Parameter name**	**Notation**
Time-dependent probability of release, given vesicle is available	*P*_r|a_(*t*)
Mean time to restore a released vesicle	τ_a_
Simulation duration	*T*
Number of pre-synaptic spikes	*K*
Time of arrival of each pre-synapticaction potential (AP)	*t*_AP, *i*_ ∈ [0, *T*], *i* = 1,2,…, *K*

In implementations of stochastic simulations it is necessary to generate random numbers from particular probability distributions. If a uniform random number generator is available, then its output, *U* ∈ (0, 1), represents a number drawn from a continuous probability distribution. Random numbers from many other distributions can be generated from uniform random numbers. For example, exponentially distributed random numbers can be obtained by the operation *T*_a_ = −τ_a_ln(*U*).

In the pseudo-code below, we assume exponentially distributed availability times as our example, but if other distributions for this random variable are desirable, then the only change required is to generate random numbers from that distribution instead.

### 4.1. Single vesicle availability and release: availability model 1

The following pseudo-code illustrates how simulations of the random variables described above can be implemented in stochastic simulations.

**Correct Implementation 1, for AM1**

Set: NextAvailabilityTime = 0
For each pre-synaptic spike, i=1:K,
             occurring at time t_i
  if t_i >= NextAvailabilityTime
    //vesicle is available
    if Pr_given_a(t_i) > unifrand()
      //Release the vesicle
      Set: LastReleaseTime = t_i
      //reset the next availability
        time, for
      //exponentially distributed
        availability times
      NextAvailabilityTime = LastReleaseTime
      +exprand(tau)
    end
  end
end
//unifrand() generates a uniformly
  distributed random number between
  0 and 1
//exprand() generates an exponentially
  distributed random number with mean tau


The pseudo-code variable **LastReleaseTime** represents our mathematical variable, *t*_s_. A direct translation of this pseudo-code into the probability that the vesicle will be released upon the arrival of AP *i*, *given t*_s_, obtains *P*_r|a_(*t*_AP, *i*_)Prob(*t*_AP, *i*_ > *t*_s_ + *T*_a_). This can be expressed as *P*_r|a_(*t*_AP, *i*_)Prob(*T*_a_ < *t*_AP, *i*_ − *t*_s_) = *P*_r|a_(*t*_AP, *i*_)*F*_*T*_a__(*t*_AP, *i*_ − *t*_s_), and thus exactly matches Equation (6), as required.

There are also several ways in which the conceptual model has been, or could be, erroneously translated into a stochastic simulation, and these are described in the following subsections.

#### 4.1.1. First incorrect implementation of availability model 1

It is stated in Scott et al. (2012) that “Following successful vesicle release, [the availability probability] is set to zero and relaxes back to 1 …” The exact form of this time changing probability [which we introduced above as *P*_a_(*t*|*t*_s_)] is expressed in Scott et al., (2012, Equation 14) as the solution to a differential equation, which has an exact solution equivalent to stating that

(12) $$P_{\text{a}}(t|t_{\text{s}})=1-\exp(-(t-t_{\text{s}})/\tau_{\text{a}}),\;\;t>t_{\text{s}},$$

where *t*_s_ was the last successful release time. Clearly Equation (12) is equivalent to Equation (1). However, it is also stated in Scott et al. (2012) that in order to create a stochastic model, “… we allowed vesicle release following comparison of” *P*_a_(*t*|*t*_s_)*P*_r|a_(*t*) “with a random number between 0 and 1.”

The following pseudo-code illustrates how this statement would be correctly implemented:

**Incorrect Implementation 1.1, for AM1**

For each pre-synaptic spike, i=1:K,
    occurring at time t_i
  Set: Pa = 1-exp(-(t_i-LastReleaseTime)/
                  tau_r)
   if Pa*Pr_given_a(t_i) > unifrand()
     //Release the vesicle
     Set: LastReleaseTime = t_i
  end
end


A direct translation of this pseudo-code into the probability that the vesicle will be released upon the arrival of the first AP after *t*_s_, at time *t*_AP, *j*_, *given t*_s_, obtains *P*_r|a_(*t*_AP, *j*_)*P*_a_(*t*_AP, *j*_|*t*_s_) which is in agreement with the correct implementation. However, this implementation also imposes a probability that the vesicle will be released upon the arrival of the second AP after *t*_s_, at time *t*_AP, *j* + 1_, as (1− *P*_r|a_(*t*_AP, *j*_)*P*_a_(*t*_AP, *j*_|*t*_s_)) × *P*_r|a_(*t*_AP, *j* + 1_) *P*_a_(*t*_AP, *j* + 1_|*t*_s_), which is the product of the probabilities of non-release at the *j*–th AP, and the calculated probability of release at the *j* + 1–th AP. This is not in agreement with Equation (6). Similar holds for the case where the vesicle is not released within the simulation after the *j* + 2–th AP, the *j* + 3–th and so forth.

The reason that the implementation is incorrect is that it does not take into account that the non-release at the *j*–th AP could have been due to release failure for an available vesicle, and this distorts the simulated probability of when the vesicle is released.

This fact might be more readily apparent by considering the following different incorrect implementation that achieves equivalent, but slightly less efficient, results:

**Incorrect Implementation 1.2, for AM1**

For each pre-synaptic spike, i=1:K,
    occurring at time t_i
  Set: Pa = 1-exp(-(t_i-LastReleaseTime)/
                  tau_r)
   if Pa > unifrand1()
     if Pr_given_a(t_i) > unifrand2()
       //Release the vesicle
       Set: LastReleaseTime = t_i
   end
end


A direct translation of this pseudo-code into the probability that the vesicle will be released upon the arrival of the first AP after *t*_s_, at time *t*_AP, *j*_ is also in agreement with the correct implementation. However, the probability that the vesicle will be released upon the arrival of the second AP after *t*_s_, at time *t*_AP, *j* + 1_, translates as [(1 − *P*_a_(*t*_AP, *j*_|*t*_s_)) + *P*_a_(*t*_AP, *j*_|*t*_s_)(1 − *P*_r|a_(*t*_AP, *j*_))] × *P*_r|a_(*t*_AP, *j* + 1_) *P*_a_(*t*_AP, *j* + 1_|*t*_s_), which is also not in agreement with Equation (6). Rearranging this gives (1 − *P*_r|a_(*t*_AP, *j*_)*P*_a_(*t*_AP, *j*_|*t*_s_)) × *P*_r|a_(*t*_AP, *j* + 1_) *P*_a_(*t*_AP, *j* + 1_|*t*_s_), which is identical to the result for the first stated incorrect pseudo-code.

Both cases of incorrect pseudo-code are incorrect because, as is clear in the second version, a random number can be drawn that is less than the probability of availability, which represents the vesicle being available. But the code does not take into account that once this happens once, there should never be a failure of availability before the vesicle is released.

The pseudo-code is equivalent to a different conceptual model where the vesicle's availability is reset to zero upon every spike arrival, regardless of whether the vesicle is released or not. Following this reset, the time until availability remains dependent on the time since the last release. This is unlike Availability Model 2, in which the reset causes the time until availability to become dependent on the time since the last spike arrival instead, and only for vesicles that are unavailable.

#### 4.1.2. Second incorrect implementation of availability model 1

A second possible incorrect implementation could result from attempting to address the problem above by implementing the following incorrect pseudo-code:

**Incorrect Implementation 2, for AM1**

Set: IsVesicleAvailable = 1
For each pre-synaptic spike, i=1:K,
    occurring at time t_i
  if IsVesicleAvailable == 0
    //vesicle is not available
    Set: Pa = 1-exp(-(t_i-LastReleaseTime)/
                  tau_r)
    if Pa > unifrand1()
      //vesicle becomes available
      IsVesicleAvailable = 1
    end
  end
  if IsVesicleAvailable == 1
    //vesicle is available
    if Pr_given_a(t_i) > unifrand2()
      //Release the vesicle
      Set: IsVesicleAvailable = 0
      Set: LastReleaseTime = t_i
  end
end


A direct translation of this pseudo-code into the probability that the vesicle will be released upon the arrival of the first AP after *t*_s_, at time *t*_AP, *j*_ is also in agreement with the correct implementation. However, the probability that the vesicle will be released upon the arrival of the second AP after *t*_s_, at time *t*_AP, *j* + 1_, translates as *P*_r|a_, if the vesicle was made available after the first spike, but not released, and as *P*_*a*_*P*_*r*|*a*_ if it became available after two spikes. When the probability of being in each of these three states is taken into account, the overall probability that the vesicle will be released upon the arrival of the second AP is *P*_r|a_(*t*_AP, *j*+1_)[*P*_a_(*t*_AP, *j*_)(1 − *P*_r|a_(*t*_AP, *j*_)) + (1 − *P*_a_(*t*_AP, *j*_))*P*_a_(*t*_AP, *j*+1_)].

As a concrete example of why this implementation is incorrect, consider an example where immediately after a vesicle release, the next arriving AP did not find a vesicle available. Suppose *P*_a_ = 0.4 at this time, and increases to *P*_a_ = 0.7 just before the next arriving spike. We should have a vesicle available after the first spike in 40% of repeated trials, and a vesicle available in 70% of repeated trials after two spikes. However, in this implementation, when the vesicle is not available after the first spike, we compare *P*_a_ = 0.7 with a random number, and 70% of the time for this case we then say a vesicle will be available after two spikes. This is incorrect, because we will have 40% of trials finding a vesicle on the first spike arrival spike and therefore by comparing *P*_a_ with 0.7 we have 100 ×(1 − 0.4) × 0.7 = 42% of trials finding a vesicle available on the second spike arrival, but not the first. Thus, there are 40 + 42 = 82% of all trials finding a vesicle available after either the first or second spike arrival. The latter value should, however, be 70%, not 82%, according to the conceptual model. Therefore, this implementation causes too many vesicles to become available by the time of the second spike arrival, if they were not available on the first arrival. The correct number to compare with a random variable upon the second spike arrival is 0.5, which would mean 30% of trials find a vesicle available on the second spike arrival, but not the first.

#### 4.1.3. Second correct implementation of availability model 1

Incorrect Implementation 2 can be corrected by changing the calculation of *P*_a_(*t*), based on Equation (2). For exponential availability times, the correction is a simple matter of replacing the pseudo-code line

Set: Pa = 1-exp(-(t_i-LastReleaseTime)/
                  tau_r)


with

Set: Pa = 1-exp(-(t_i-t_(i-1))/tau_r)


For non-exponentially distributed arrival times, the required change is more complex, but readily follows in a similar fashion, from Equation (2).

#### 4.1.4. Third correct implementation of availability model 1, for exponential availability times

We stated above that for the special case of exponentially distributed times for a vesicle to become available, Availability Model 1 is equivalent to a conceptual model where a vesicle becomes available upon generation of the next event within a Poisson process with rate 1/τ_a_, following release, as in Loebel et al. (2009). An implementation of this conceptual model is illustrated in the following pseudo code, where it is assumed that the Poisson events had previously been calculated, and that NextPoissonTime(x) is a function that returns the time of the Poisson event immediately following the time given by its argument.

**Correct Implementation 3, for AM1**

Set: NextAvailabilityTime = 0
For each pre-synaptic spike, i=1:K,
     occurring at time t_i
  if t_i >= NextAvailabilityTime
    //vesicle is available
    if Pr_given_a(t_i) > unifrand()
      //Release the vesicle
      Set: NextAvailabilityTime
         = NextPoissonTime(t_i)
    end
  end
end



### 4.2. Single vesicle availability and release: availability model 2

The following pseudo-code illustrates how simulations of Availability Model 2 can be implemented in stochastic simulations. Note that the only difference in comparison with the pseudo-code for Availability Model 1 is that the time of next availability (for unavailable vesicles only) is dependent only on the last spike arrival time, not the last release time, in order to match the conceptual model.

**Correct Implementation 1 for AM2**

Set: NextAvailabilityTime = 0
For each pre-synaptic spike, i=1:K,
   occurring at time t_i
  if t_i >= NextAvailabilityTime
    //vesicle is available
    if Pr_given_a(t_i) > unifrand()
      //Release the vesicle and reset the
        next availability time, for
      // exponentially distributed
         availability times
      NextAvailabilityTime = t_i
                    +exprand(tau)
    end
  else
    //vesicle is unavailable; reset the
      next availability time, for
    //exponentially distributed
      availability times
    NextAvailabilityTime = t_i +exprand(tau)
  end
end



A direct translation of this pseudo-code into the probability that the vesicle will be released upon the arrival of AP *i*, given that it was not released by the time of AP *i* − 1, obtains *P*_r|a_(*t*_AP, *i*_)Prob(*t*_AP, *i*_ > *t*_AP, *i* − 1_ + *T*_a_). This can be expressed as *P*_r|a_(*t*_AP, *i*_)Prob(*T*_a_ < *t*_AP, *i*_ − *t*_AP, *i* − 1_), and thus exactly matches Equation (7), as required, upon substitution of Equation (5).

It is possible to incorrectly implement Availability Model 2 in a manner directly analogous to that in the first incorrect implementation of Availability Model 1. However, an implementation analogous to the second incorrect implementation of Availability Model 1, will actually be correct for Availability Model 2, since now the probability of availability is dependent only on the last AP arrival time.

### 4.3. Multiple trials of single vesicle availability and release

#### 4.3.1. Availability model 1 with exponential availability times

For the special case of exponentially distributed availability times, for each trial in which a vesicle is unavailable at the previous AP, the probability of becoming available by the current one will be identical for each trial (provided the input APs occur at the same times in all trials). As a direct consequence of this, the probability that *w* trials result in a vesicle becoming available, out of *v* in which a vesicle was not available at time *t*_AP, *i*_, is given by the binomial distribution, as mentioned and studied numerous times, e.g., (Vere-Jones, 1966; Melkonian and Kostopoulos, 1996; Quastel, 1997; Matveev and Wang, 2000b; Pfister et al., 2010; Reich and Rosenbaum, 2013). We introduce a random variable, *W*, to describe the number of unavailable vesicles that become available. We have

$$\text{Prob}(W=w|v)=\left(\begin{array}{c} v\\ w \end{array}\right){\left(1-P_{\text{a}}\right)}^{\left(v\;-w\right)}{\left(P_{\text{a}}\right)}^{w}.$$

That this expression holds enables a stochastic simulation implementation that is far more efficient than repeating each of *Z* trials independently, as described in the following pseudo-code.


Set: NumUnavailable = 0
For each pre-synaptic spike, i=1:K,
    occurring at time t_i
  //Calculate probability of availability
    at t_i for any unavailable vesicles
  Set: Pa = 1-exp(-(t_i-t_(i-1))/tau_r)
  //calculate the number to become
    available by t_i
  Set: NumUnavailable = NumUnavailable
     − binornd(NumUnavailable,Pa);
  //calculate number to release at t_i
  Set: NumUnavailable = NumUnAvailable
     + binornd(NumTrials-NumUnavailable,
       Pr_given_a(t_i))
end
//binornd(v,w) calculates a binomially
  distributed random number with
// a maximum value of v, and mean vw.


This algorithm is an extension of an algorithm presented by Quastel (1997) (see also Pfister et al., 2010) for the case where *P*_a_ is time-independent.

In the above pseudo-code, we have calculated two independent binomially distributed random numbers for each pre-synaptic AP arrival. The second random number describes the number of trials in which an available vesicle is released. This is accurate with respect to both Release Model 1 and Release Model 2 under the assumptions of this paper, since the simulation calculated how many trials have a vesicle available at each time *t*_AP, *i*_, and the probability of release is independent and identical for all trials in both release models. Mathematically, if we denote the random variable describing the number of vesicles released as *U*, when *s* are available, we have

$$\text{Prob}(U=u|s)=\left(\begin{array}{c} s\\ u \end{array}\right){\left(1-P_{\text{r}|\text{a}}\right)}^{\left(s\;-u\right)}{\left(P_{\text{r}|\text{a}}\right)}^{s}.$$

The use of binomially distributed random numbers in this way will not be correct for a possible alternative release models where the probability of release, given availability, depends on the history of vesicle release in each trial, because the refill events are not independent in that case [see, e.g., Quastel (1997) for mathematical analysis of this case].

#### 4.3.2. Availability model 2 with exponential availability times

The binomial approach described above for the special exponential case of Availability Model 1, will also correctly simulate Availability Model 2 with exponential availability times, since, as discussed above, the two models are equivalent under this special case.

#### 4.3.3. Availability models 1 and 2 with non-exponential availability times

The algorithm above holds only for exponential availability times, as it relies on the fact that in this case *P*_a_,1(*t*|*t*_s_) = 1− exp(−(*t* − *t*_s_)/τ_a_), *t* ≥ *t*_s_ for all vesicles. For non-exponential availability times, the number of vesicles unavailable due to release from all previous spikes needs to be tracked, and consequently many more binomial random numbers need to be generated following each AP. Moreover, *P*_a_(*t*) needs to be calculated using Equation (2).

### 4.4. Comparison of algorithm implementation efficiencies

We have aimed in the pseudo-code implementations above to describe computationally efficient algorithms that require as few random numbers to be generated as possible.

We note that the implementation suggested, for example, in Loebel et al. (2009) [see also Sterratt et al., (2011, p. 188)] involves an accurate approximation of a true Poisson point process, and this approximation is particularly relevant to any simulation in which time is discretised into uniform intervals of Δ*t*, such as in most simulations that involve numerical solution of differential equations. The well-known approximation states that provided that Δ*t* « τ_a_, a Poisson point process event occurs within any given time interval of duration Δ*t* with probability $\frac{\Delta t}{\tau_{\text{a}}}$.

A stochastic simulation based on this approximation requires comparison of $\frac{\Delta t}{\tau_{\text{a}}}$ with a uniform random number at every time step of the simulation between times 0 and *t*_*K*_. It is possible to alter the implementation so that the Poisson events are only calculated during the simulation, rather than prior, where a comparison of a uniform random number with $\frac{\Delta t}{\tau_{\text{a}}}$ is carried out for every time step following vesicle release, until a random number is generated that is larger than $\frac{\Delta t}{\tau_{\text{a}}}$.

However, such implementations are potentially very inefficient, because many random numbers must usually be generated for every unavailable vesicle, whereas only one random number need be generated in, for example, Correct Implementation 1 for AM1.

## 5. Examples: comparing stochastic simulation implementations

### 5.1. Errors in simulating probability of release, and mean number of releases after *K* spike arrivals, for exponential availability times

We consider a scenario where pre-synaptic APs arrive at a synapse periodically with frequency *f* Hz. We consider *Z* repeated trials following an initial condition where a vesicle is assumed to have just been released, in all trials, at the start of our simulations. We calculate the number of trials in which the next vesicle release occurs after the first spike, the second spike and so forth. We obtain results for *f* between 5 and 150 Hz, for *Z* = 100,000, τ_a_ = 0.5 s and *K* = 50 pre-synaptic spikes (as a maximum; the simulation stops when a vesicle is first released). Thus, the AP times are *t*_AP, *i*_ = *i*/*f*, *i* = 1, 2, …,50.

We estimated the probability that the vesicle was next released after *i* = 1, 2, … 20 APs following vesicle release at time *t* = 0, by evaluating the fraction of trials in which the vesicle was first released after the *i*–th spike. We then calculated the absolute value of the difference in the estimated probability for several correct and incorrect implementations, and also the relative error, relative to the correct version.

In order to clarify the significance of the values we obtained for absolute and relative error, we also considered a simulation where *H* = 100 spikes per trial periodically arrive with frequency *f*, and for each implementation counted the total number of vesicles released as a function of *f*. We then compared the mean number released after *H* spikes, calculated from *Z* = 10,000 repeats of each implementation, as well as the maximum and minimum numbers released.

Finally, in order to show that our simulations and mathematical analysis is correct for Availability Model 1 for both periodic and non-periodic AP arrivals, we compare correct and incorrect implementations for each case with results predicted by the Equations (9) and (10).

#### 5.1.1. Results for availability models 1 and 2 with release model 1

**Figure 1:**
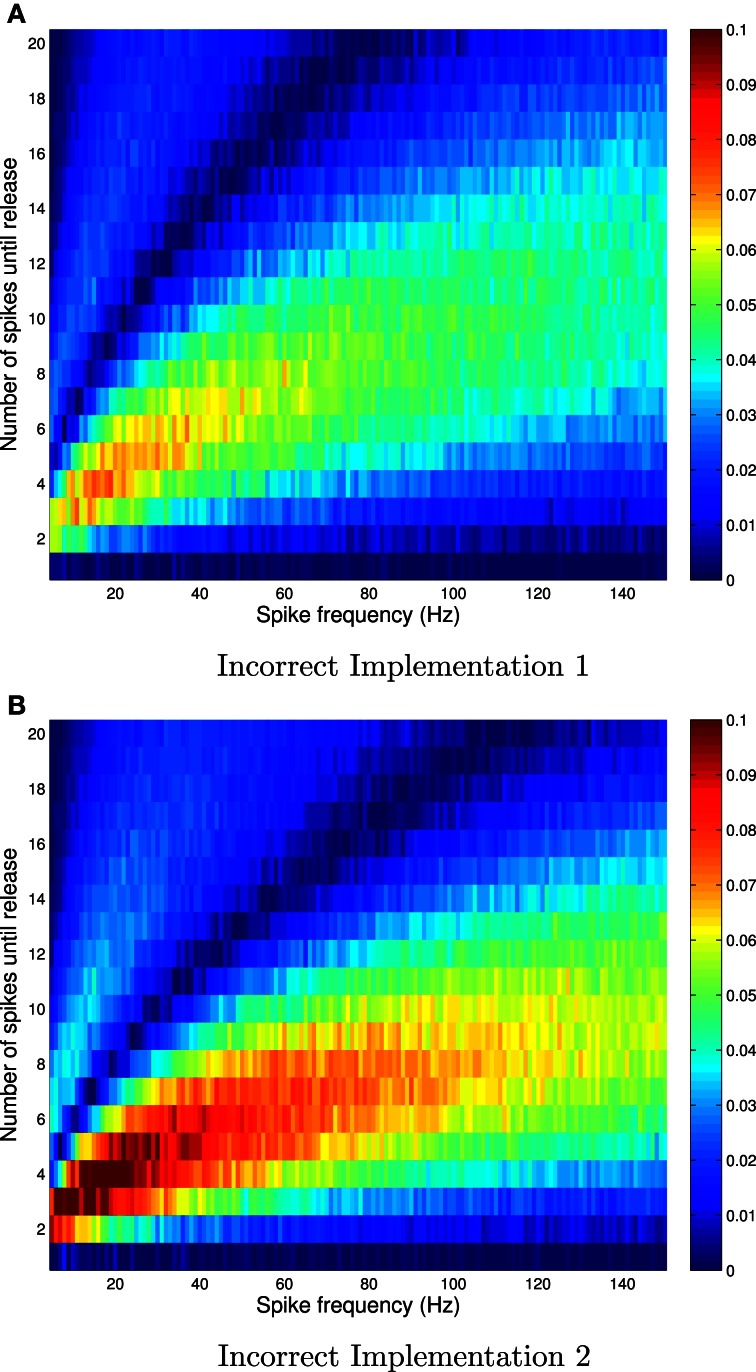
**Absolute errors for incorrect implementation 1 (A) and incorrect implementation 2 (B), for Availability Model 1, with exponentially distributed availability times.** The data was obtained by empirically estimating the probability of release after *i* spikes, as a function of the frequency of periodically arriving pre-synaptic action potentials, by stochastically simulating *Z* = 100,000 trials for each condition. The absolute error can be as high as 0.1, and higher errors occur at low frequencies.

**Figure 2:**
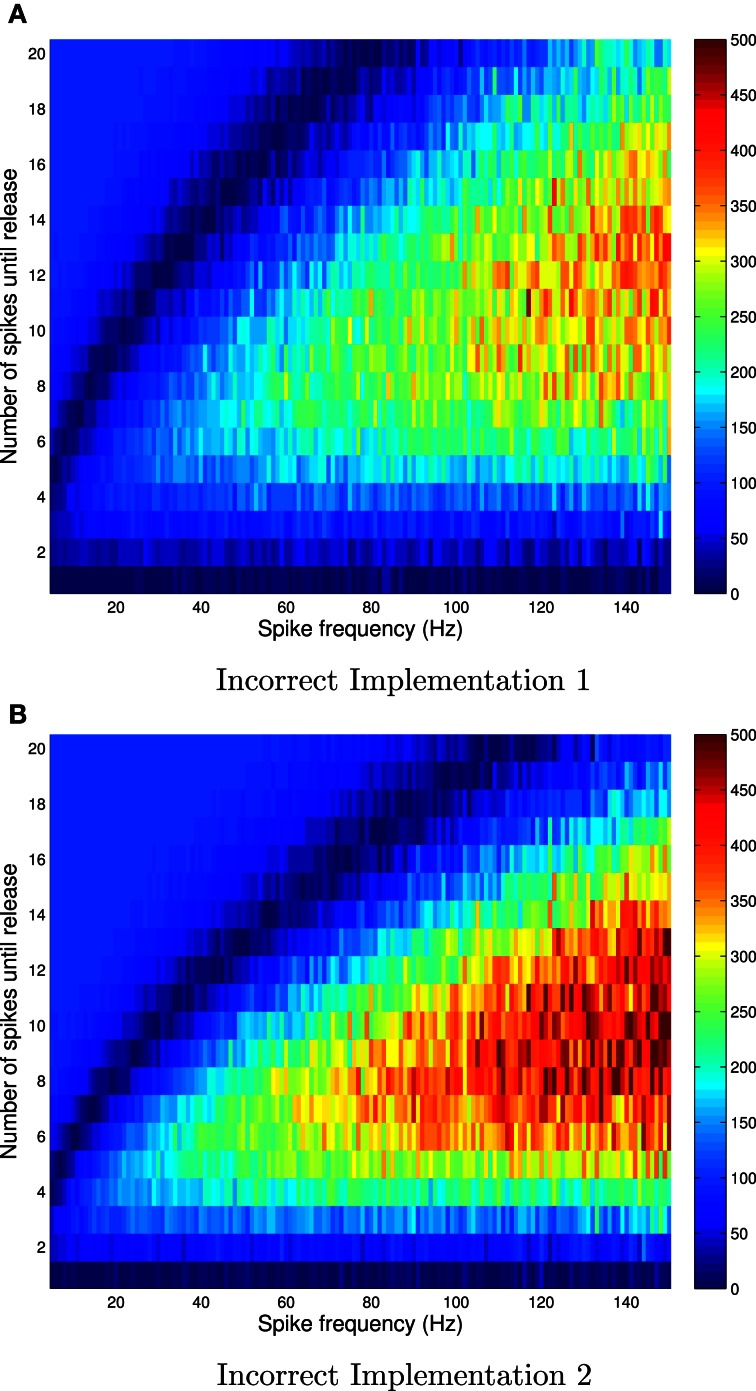
**Relative errors for incorrect implementation 1 (A) and incorrect implementation 2 (B) for Availability Model 1, with exponentially distributed availability times.** The data was obtained by empirically estimating the probability of release after *i* spikes, as a function of the frequency of periodically arriving pre-synaptic action potentials, by stochastically simulating *Z* = 100,000 trials for each condition. The largest relative errors occur for higher frequencies.

We set the probability of release, given availability to *P*_r|a_ = 0.6. Figure 1 shows the absolute error, and Figure 2 shows the relative error between the correct and incorrect implementations, for Availability Model 1.

The absolute error, as predicted by the theory, is zero after the first pre-synaptic spike, for all *f*. However, it is clear that the absolute error can be as high as 10% for subsequent spikes, and is highest for low frequencies. It is also clear that the relative error can be very high for high frequencies. In these cases, the probability of release is relatively small for all subsequent spikes, and hence the absolute error is low. Yet the relative error can be higher than 500% at *f* = 150 Hz.

**Figure 3:**
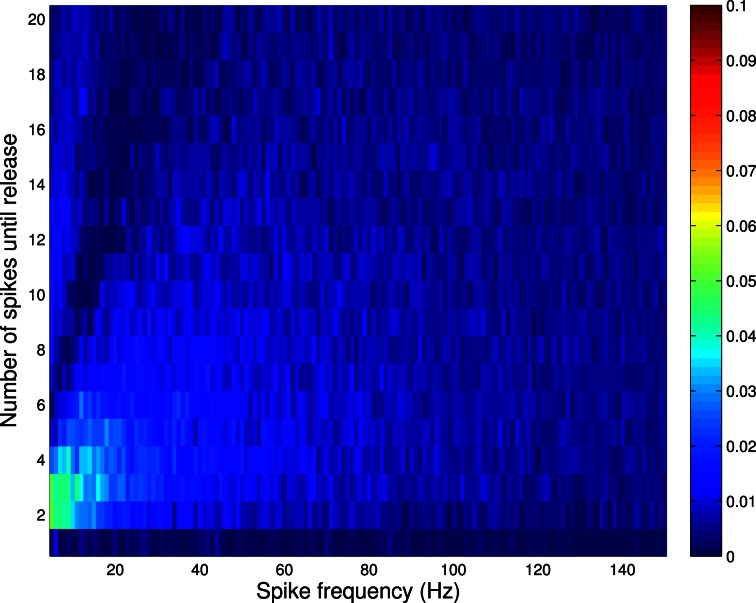
**Absolute error between correct and incorrect implementation for Availability Model 2, with exponentially distributed availability times.** The data was obtained by empirically estimating the probability of release after *i* spikes, as a function of the frequency of periodically arriving pre-synaptic action potentials, by stochastically simulating *Z* = 100,000 trials for each condition. The largest error occurs for low frequencies, but is much smaller than for Availability Model 1.

Figure 3 shows the absolute error between the correct and incorrect implementation for Availability Model 2 (recall from above that an implementation analogous to the second incorrect implementation of Availability Model 1, is correct for Availability Model 2). The incorrect implementation clearly shows a smaller error than for Availability Model 1.

**Figure 4:**
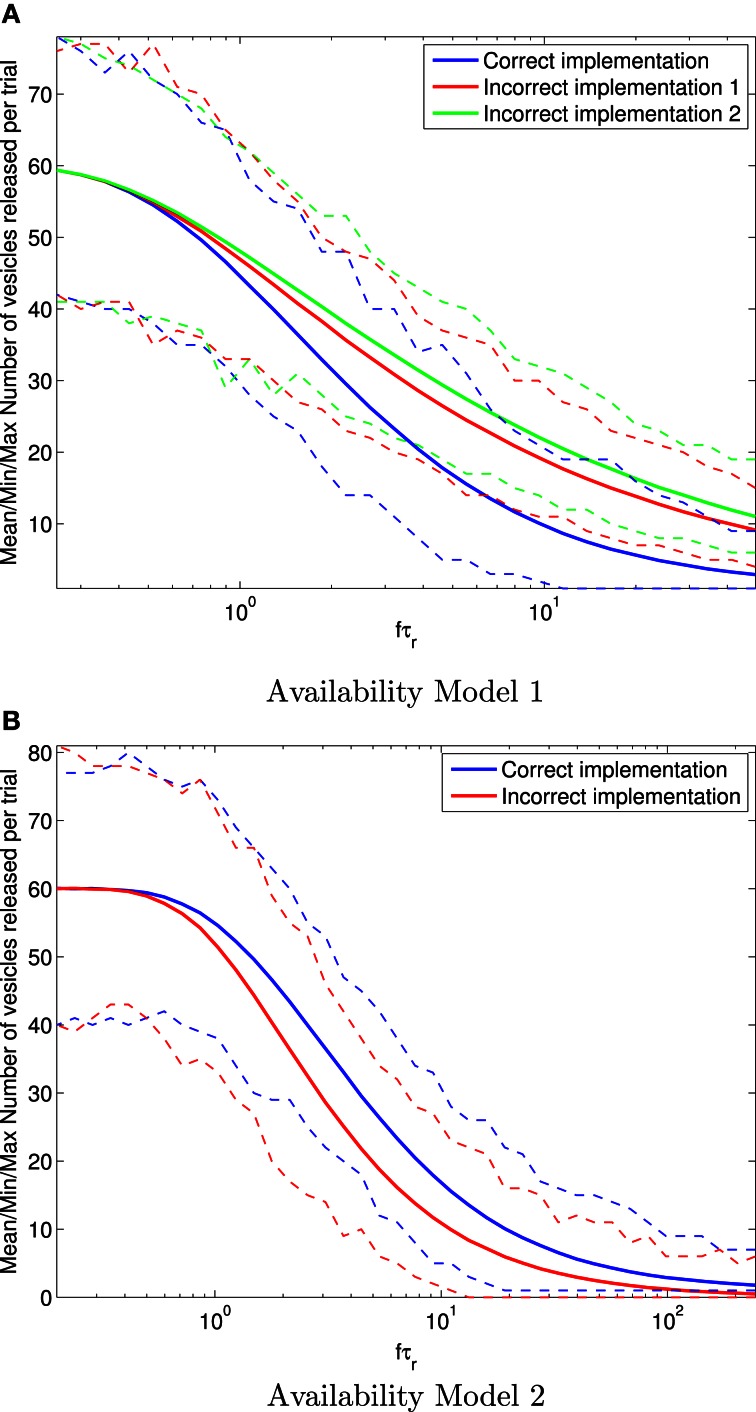
**Mean (solid traces) number of vesicles released in total after 100 periodic pre-synaptic action potential arrivals for Availability Model 1 (A) and Availability Model 2 (B).** The minimum and maximum number of vesicles released are shown with (dashed traces). For each frequency f, all statistics are derived from 10,000 stochastic simulations. Clearly the incorrect implementations can over or under estimate the correct number of vesicles released.

Results for the mean number of vesicles released after *H*= 100 spikes are shown in Figure 4. It is clear for Availability Model 1 that the mean number of vesicles released per trial of 100 spikes becomes more inaccurate for the incorrect models as *f* τ_r_ increases. For example, at *f*τ_r_ > 10, the incorrect models can produce more than twice as many vesicles as the correct one. It is clear for Availability Model 2 that the mean number of vesicles released per trial of 100 spikes is inaccurate for the incorrect model, similar to Availability Model 1. However, now the incorrect model underestimates the number of vesicles released, whereas for Availability Model 1, the incorrect models overestimated this number.

The data in Figure 4 also shows that all models correctly produce a mean of *P*_r|a_ = 0.6 vesicles released at low frequencies, where the availability always has time to recover to close to 100%.

**Figure 5:**
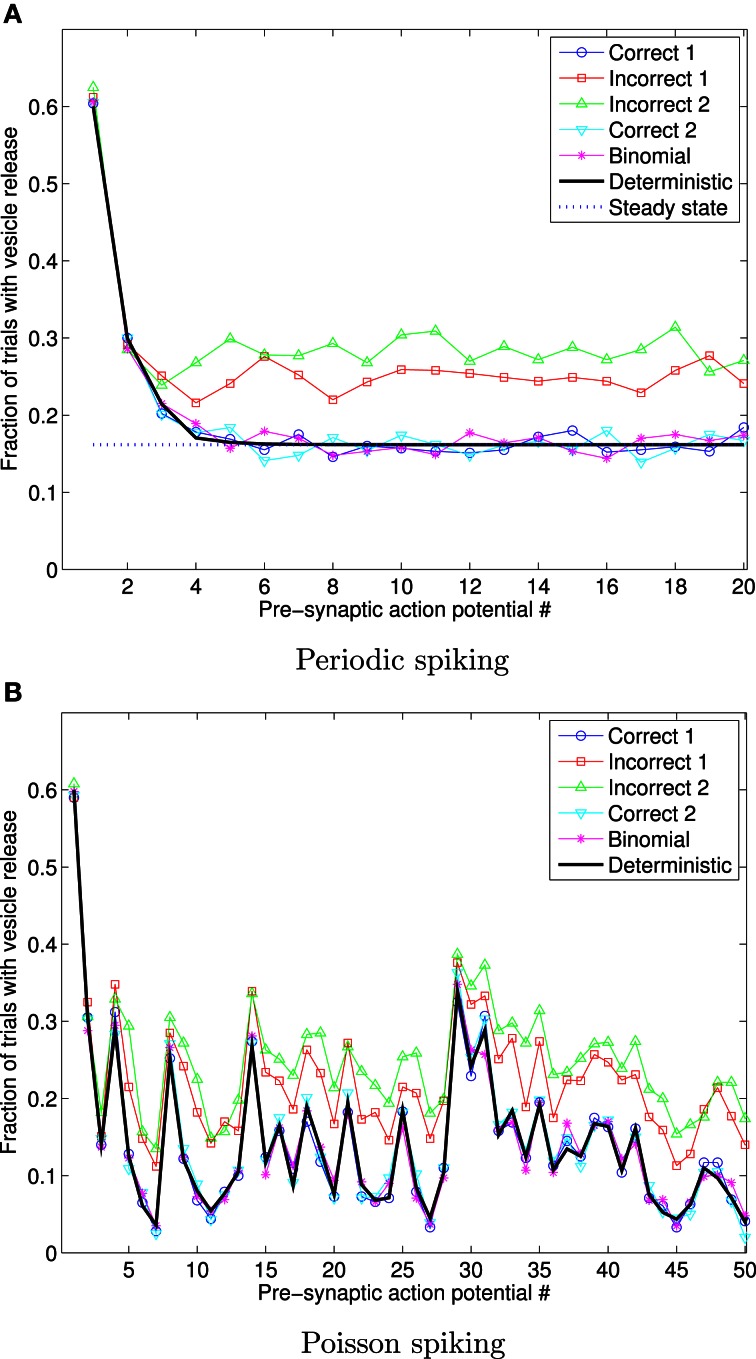
**Fraction of 1000 trials in which vesicles are released, for each of a sequence of 20 periodic spikes (A), and 50 Poisson spikes (B), and vesicles with exponentially distributed availability times.** The frequency in both cases is 10 Hz. The traces for **Deterministic**, and **Steady state** were obtained using Equations (9) and (10). This data shows that the incorrect implementations give markedly different outcomes to the correct stochastic simulation implementations, and to the deterministic expression for the mean number of trials in which vesicles are released.

Figure 5 shows the fraction of 1000 trials in which vesicles are released in response to a sequence of 20 periodically arriving APs, with frequency 10 Hz, and to a sequence of 50 APs arriving at times corresponding to a Poisson point process, with mean frequency 10 Hz. In this figure, the data for the **Deterministic**, and **Steady state** cases were obtained using Equations (9) and (10), respectively (derived previously in the literature, as stated and referenced above) and clearly match the correct stochastic simulations.

#### 5.1.2. Release model 2

In order to demonstrate how to incorporate a time dependent release probability, we consider a standard model of facilitation (see, e.g., Scott et al., 2012). The change in release probability can be expressed as a differential equation, but it is clearer to write a piecewise equation as follows:

$$\begin{array}{l} \;P_{\text{r}|\text{a}}(t)=Q,\;\;t<t_{\text{AP},1},\\ P_{\text{r}|\text{a}}(t_{\text{AP},i})=P_{\text{r}|\text{a}}(t_{\text{AP},i}^{-})+S(1-P_{\text{r}|\text{a}}(t_{\text{AP},i}^{-})),\;t=t_{\text{AP},i},\\ \;P_{\text{r}|\text{a}}(t)=Q-(Q-P_{\text{r}|\text{a}}(t_{\text{AP},i}))\;t\in [t_{\text{AP},i},t_{\text{AP},i\;+1}),\\ \;\exp\left(-(t-t_{\text{AP},i})/\tau_{\text{f}}\right) \end{array}$$

where *Q* is a parameter that describes the steady-state release probability, when there have been no arriving APs for a long time, and *S* is a parameter that describes the fractional increase (relative to the maximum possible increase) in release probability that occurs for every arriving pre-synaptic AP. We also have a time constant of facilitation, τ_f_, which determines how quickly the release probability decays back to its resting value, *Q*. Examples of appropriate parameters might be *Q* = 0.4, and *S* = 0.2, similar to Scott et al. (2012).

Note that this particular function *P*_r|a_(*t*) is determined entirely once the sequence of pre-synaptic spikes is known, and consequently it is easily incorporated into the stochastic simulation algorithms described above, and we do not show example results here.

The same observations hold for release-independent depression with frequency-dependent recovery, in which case τ_f_ can also change with time (Fuhrmann et al., 2004; Scott et al., 2012).

### 5.2. Comparison of availability models 1 and 2 for non-exponential availability times

In order to demonstrate that a non-exponential availability model provides different outcomes for Availability Models 1 and 2, we consider the case of Rayleigh distributed availability times, with mean τ_a_ = 0.5 s.

**Figure 6:**
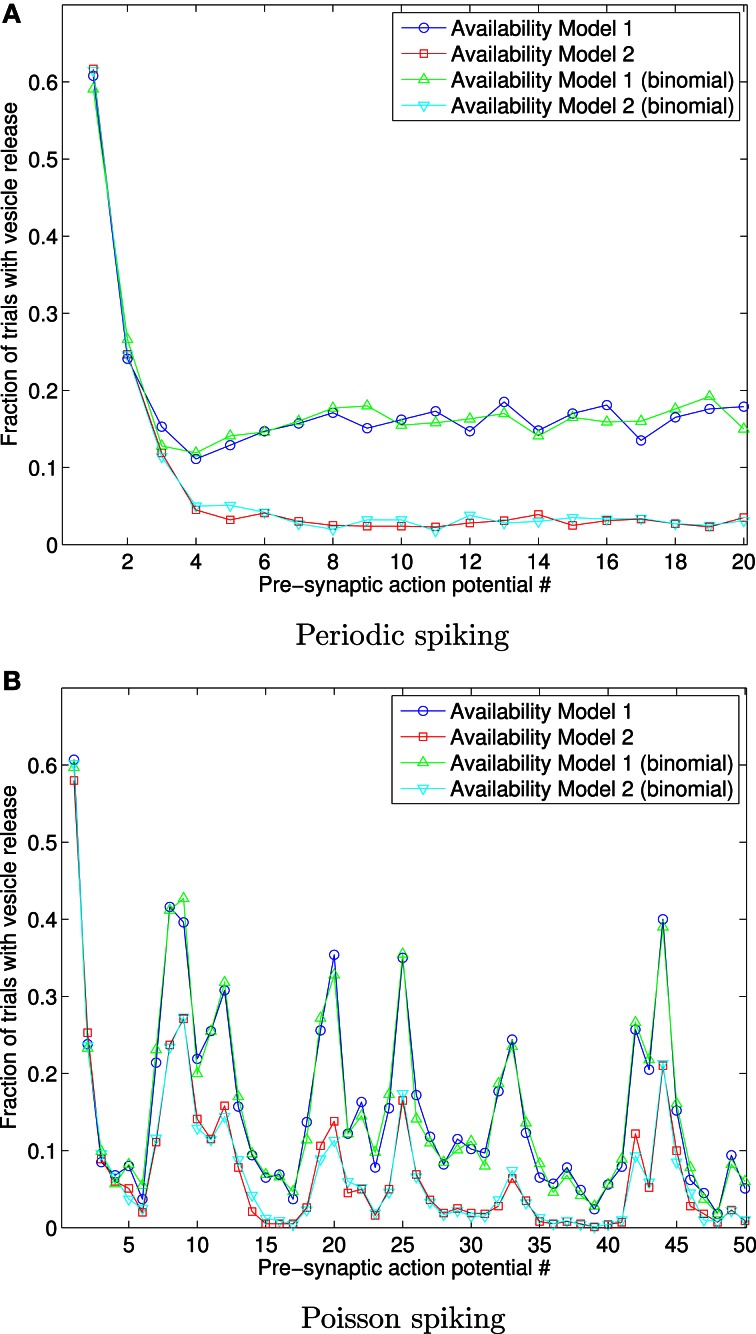
**Fraction of 1000 trials in which vesicles are released, for each of a sequence of 20 periodic spikes (A), and 50 Poisson spikes (B), and vesicles with Rayleigh distributed availability times.** The frequency in both cases is 10 Hz. This data shows that Availability Models 1 and 2 provide markedly different outcomes for Rayleigh distributed availability times, unlike the identical outcomes for exponentially distributed times. The data also shows that extension of the binomial approach matches the data where each trial is individually simulated.

Figure 6 shows the fraction of 1000 trials in which vesicles are released, for both availability models, in response to a sequence of 20 periodically arriving APs, with frequency 10 Hz, and to a sequence of 50 APs arriving at times corresponding to a Poisson point process, with mean frequency 10 Hz.

The results shown in Figure 6 were obtained both by a direct adaptation of the correct stated pseudo-code above to Rayleigh distributed availability times, and also by direct adaption of the binomial approaches described, for Availability Model 2. The binomial results for Availability Model 1 required a more complex algorithm, where the number of vesicles unavailable due to release from all previous spikes needed to be tracked, and consequently many more binomial random numbers generated than for Availability Model 1. The data can be seen to match in either implementation, but to be quite different for each Availability Model.

## 6. Conclusions and extensions

### 6.1. Correct and efficient stochastic simulations of short-term plasticity

We have shown that various correct implementations of a stochastic simulation of either Availability Model 1 or 2 are possible. However, it is also possible to incorrectly implement either model. For Availability Model 1, two kinds of incorrect implementation result in more vesicle releases than should be the case. For Availability Model 2, an incorrect implementation results in less vesicle releases than should be the case.

We have also shown that some correct implementations are more efficient than others. In particular, we first stated an implementation that requires only a single random number to be generated each time a vesicle is released. This is more efficient than an implementation based on generation of a Poisson process that determines availability times, and much more efficient than generating a random number for every time step in a simulation.

We also have shown that when multiple independent vesicle releases are considered, the most efficient stochastic simulation implementation can be achieved by generating binomial random numbers.

### 6.2. Consequences of equivalence of availability models only for exponential availability times

We have discussed that the two availability conceptual models are equivalent when the availability times are exponentially distributed, and this can be derived as a consequence of a well known property of a homogeneous Poisson point process. When the availability times are non-exponential, the two availability conceptual models we consider are generally non-equivalent.

As we have shown, these points are important in terms of their consequences for the implementations that can be used to correctly simulate Availability Model 1. Another consequence is that the popular differential equation approach to describing the mean number of available vesicles could have analogous correct simple forms for Availability Model 2, but not for Availability Model 1.

### 6.3. Binomial-based stochastic simulations for non-exponential availability times

When *T*_a_ is not exponentially distributed, no simple adaptation of the binomial approach will work for Availability Model 1, because there is no simple procedure for calculating random numbers corresponding to the random variable *W*. However, it is possible to keep track of how many vesicles were made unavailable by each AP, and how many are restored by the time of each subsequent AP, and perform a stochastic simulation that calculates as many binomial random numbers as there have been prior APs for which unavailable vesicles still exist. This procedure must make use of Equation (2) to calculate the probability of availability by the time of the next spike, using the time of the previous spike.

Such an algorithm may be more efficient than independently simulating each trial, provided that APs arrive relatively slowly compared with the mean time for a released vesicle to become available. Figure 6 shows that an implementation of this approach for Availability Model 1 and Rayleigh distributed available times agrees with data from an approach that individually simulates each trial.

For Availability Model 2, the binomial approach will work for arbitrary *F*_*T*_a__, since the probability of an unavailable vesicle becoming available will be the same for all trials, as was the case for the data in Figure 6. Moreover, only the cumulative distribution function of the availability times need be known to carry this out, whereas Equation (2) needs to be computed for Availability Model 2. Such an algorithm is a straightforward extension of an algorithm described by Quastel (1997) for constant probabilities for any released vesicle to be available by the next spike.

### 6.4. Other models

We have considered only the simplest models in this paper. Other more complex models of availability and release have been proposed. For example, the mean times to availability for a vesicle may change over time (Wong et al., 2003), vesicles may also be released spontaneously in the absence of pre-synaptic APs (Sterratt et al., 2011), and multiple vesicles may be readily available for release at any site (although in this model only one can be released per pre-synaptic spike) (de la Rocha and Parga, 2005). Stochastic simulations that faithfully reflect these models can be readily devised by extension of the algorithms presented in this paper.

### Conflict of interest statement

The authors declare that the research was conducted in the absence of any commercial or financial relationships that could be construed as a potential conflict of interest.
